# Common brain activation patterns in schizophrenia patients with auditory verbal hallucinations: A conjunction analysis

**DOI:** 10.1192/j.eurpsy.2025.1662

**Published:** 2025-08-26

**Authors:** L. Barbosa, P. Fuentes-Claramonte, P. Salgado-Pineda, F. Neuhaus, P. Del Olmo, B. Hoyas-Galán, P. McKenna, E. Pomarol-Clotet

**Affiliations:** 1 FIDMAG Germanes Hospitalàries Research Foundation; 2 CIBERSAM, ISCIII, BARCELONA; 3 Hospital Benito Menni, CASM, GRANOLLERS, Spain

## Abstract

**Introduction:**

Auditory verbal hallucinations (AVH) are one of the primary symptoms of schizophrenia, but the biological mechanisms underlying them remain uncertain (1,2). Theoretical approaches have proposed that AVH are caused by abnormal activity in the auditory cortex; or that they represent misinterpreted cognitive activity such as inner speech. Recently, our group found, using a symptom capture task, that AVH did not trigger activity in the auditory cortex, but instead in language-related areas, thus shifting the focus towards cognitive theories of AVH (3). To date, cognitive approaches have only been preliminarily investigated, and mostly in psychological studies (1,2).

**Objectives:**

Our aim was to test the theory that a disturbance in inner speech processes underlie AVH. We used conjunction analysis to examine common activation patterns between the experience of AVH and phonological encoding.

**Methods:**

Eleven patients meeting DSM-5 criteria for schizophrenia or schizoaffective disorder with near-continuous AVH underwent fMRI during symptom capture and during a phonological encoding task. In the symptom capture task, the patients were instructed to press their left index finger when they begin to hear an AVH, wait three minutes, mentally repeat what they heard, and then press their right index finger. The phonological encoding task required them to indicate, via button press, whether the names of two objects shown in line drawings rhymed.

Pre-processing and analyses were carried out with FSL software using linear models. Activation maps were thresholded at p<0.05, cluster-corrected for multiple comparisons. To find regions of common activation between the two tasks, the activation maps from the contrasts of interest were binarized and entered into a conjunction analysis. Regions showing significant activation in both tasks simultaneously were considered activated in the conjunction analysis.

**Results:**

The conjunction analysis showed common activation in several regions involved in phonological encoding, such as Broca’s area and its right homologue, supplementary motor area bilaterally, Wernicke’s area and cerebellum, in patients with AVH.

**Conclusions:**

These results support a non-perceptual origin of AVH and link them to brain areas related to the phonological loop and working memory in schizophrenia.

**Disclosure of Interest:**

None Declared

